# Supplementary material for: Harnessing Phosphorous (P) Fertilizer-Insensitive Bacteria to Enhance Rhizosphere P Bioavailability in Legumes
Source: Microorganisms. 2024 Feb 8;12(2):353. doi: 10.3390/microorganisms12020353 (PMC10892362; doi:10.3390/microorganisms12020353)
Supplement: Supplementary file 1 [file microorganisms-12-00353-s001.zip › microorganisms-2834708-supplementary.pdf]

microorganisms

Supplementary Materials

Table S1. Log2 fold changes (Log2FC) of microbial abundance mean from unfertilized soil in common beans. Log fold change was calculated by subtracting the baseMean counts of log ratios of each microbial taxa at the species level; FDR< 0.05. Enrich group represents the plant variety in which the corresponding bacteria species is in greater abundance. padj represents the FDR adjusted *p*-value.

Common beans Anasazia vs Cowboy

Bacteria	Enrich group	Log2 fold changes (Log2FC)	padj	Function
Mobilisporobacter senegalensis	Anasazi	18.24406755	6.97E-13	*phoA-phoD*
Desnuesiella massiliensis	Anasazi	20.86322716	8.55E-11	
Pseudomonas oryzae	Anasazi	20.36681739	9.02E-11	*PSB*
Sporomusa sphaeroides	Anasazi	20.50977553	2.03E-10	*phoA-phoD*
Anaerotaenia torta	Anasazi	19.87477933	1.53E-08	*phoA-phoD*
Parasegetibacter luojiensis	Anasazi	17.32077301	1.73E-08	*PSB*
Pseudomonas sagittaria	Anasazi	19.00574355	3.98E-08	*PSB*
Lutispora thermophila	Anasazi	18.85559879	1.18E-07	
Pseudomonas oleovorans	Anasazi	18.82500478	2.79E-07	*PSB*
Pseudomonas resinovorans	Anasazi	18.69856922	2.69E-06	*PSB*
Anaerospora hongkongensis	Anasazi	20.46654823	2.69E-06	*phoA-phoD*
Hungateiclostridium cellulolyticum	Anasazi	19.52520814	2.77E-06	
Phaeospirillum molischianum	Anasazi	19.20475576	1.28E-05	
Anaerobacterium chartisolvens	Anasazi	18.27072606	7.89E-05	
Lacrimispora saccharolytica	Anasazi	19.36907668	0.00012071	
Ruminiclostridium cellulolyticum	Anasazi	18.36482819	0.00034705	
Ruminiclostridium hungatei	Anasazi	20.20184081	0.00836473	
Clostridium nitrophenolicum	Anasazi	18.84269744	0.00943624	
[Desulfobacterium] catecholicum	Anasazi	18.9649193	0.02331291	
Aliterella antarctica	Cowboy	-22.93163735	4.10E-10	
Cylindrospermum stagnale	Cowboy	-19.7866546	1.45E-08	
Oscillatoria acuminata	Cowboy	-22.51589679	2.58E-07	
Flavisolibacter tropicus	Cowboy	-1.093165167	9.50E-05	
Legionella cherrii	Cowboy	-5.931627693	0.00195883	
Planktothrix spiroides	Cowboy	-23.4503348	0.00292182	
Paenibacillus endophyticus	Cowboy	-1.682837648	0.01412165	
Bacillus mediterraneensis	Cowboy	-4.967865548	0.01925045	
Paenibacillus algorifonticola	Cowboy	-4.186432429	0.02331291	

Common beans Anasazia vs Black Beard

Bacteria	Enrich group	Log2 fold changes (Log2FC)	padj	Function
Mobilisporobacter senegalensis	Anasazi	17.46866845	1.23E-11	*phoA-phoD*
Desnuesiella massiliensis	Anasazi	20.64184843	1.28E-10	
Clostridium tunisiense	Anasazi	20.13921923	1.28E-10	
Anaerotaenia torta	Anasazi	19.68640012	3.38E-08	*phoA-phoD*
Cyanothece sp. PCC 7425	Anasazi	16.82850704	4.09E-08	
Clostridium swellfunianum	Anasazi	18.40798121	4.09E-08	
Lutispora thermophila	Anasazi	18.62167774	2.56E-07	
Sunxiuqinia rutila	Anasazi	20.28304711	6.88E-07	
Sunxiuqinia faeciviva	Anasazi	20.4421622	3.69E-06	
Hungateiclostridium cellulolyticum	Anasazi	19.29404828	5.47E-06	
Phaeospirillum molischianum	Anasazi	18.98297814	2.38E-05	
Anaerobacterium chartisolvens	Anasazi	18.03640105	0.00014384	
Ruminiclostridium cellulolyticum	Anasazi	18.16040593	0.00067133	
Asticcacaulis endophyticus	Anasazi	17.21907478	0.00251354	*phoA-phoD*
Anaerosporobacter mobilis	Anasazi	21.97335007	0.01038019	
Azospirillum brasilense	Anasazi	2.261040436	0.01202777	*PSB*
Ruminiclostridium hungatei	Anasazi	19.98751551	0.01202777	
Clostridium nitrophenolicum	Anasazi	18.61473215	0.01355384	
[Desulfobacterium] catecholicum	Anasazi	18.73239433	0.03222814	
Herbinix luporum	Anasazi	5.599646619	0.03972306	*phoA-phoD*
Cylindrospermum stagnale	Black Beard	-20.31640439	5.36E-09	
Aliterella antarctica	Black Beard	-21.8449055	5.36E-09	
Paenibacillus oenotherae	Black Beard	-24.37144045	6.88E-07	
Legionella cherrii	Black Beard	-6.008188756	0.0023753	
Paenibacillus pocheonensis	Black Beard	-3.237119606	0.00279293	
Rhizobacter gummiphilus	Black Beard	-2.298062264	0.00301638	
Paenibacillus frigoriresistens	Black Beard	-5.316563348	0.01883372	
Cohnella lupini	Black Beard	-3.992174423	0.025788	
Verrucomicrobium spinosum	Black Beard	-1.940968607	0.03222814	
Planktothrix spiroides	Black Beard	-19.58112484	0.03709835	

Table S2. Log2 fold changes (Log2FC) of microbial abundance mean from unfertilized soil in cowpeas. Log FC was calculated by subtracting the baseMean counts of log ratios of each microbial taxa at the species level; FDR< 0.05. Enrich group represents the plant variety in which the corresponding bacteria species is in greater abundance. padj represents the FDR adjusted *p*-value.

Bacteria	Enrich group	Log2 fold changes (Log2FC)	padj	Function
Sporichthya polymorpha	Cal#5	-6.8341689	9.12E-05	
Paenibacillus oenotherae	Cal#5	-22.607057	0.00010675	*phoA*
Microlunatus speluncae	Cal#5	-5.9317786	0.01144082	*phoA*
Hyphomicrobium hollandicum	Cal#5	-5.9181764	0.0300987	*PSB*
Arenimonas daejeonensis	Cal#5	-6.9411873	0.02432705	*PHO*
Oscillatoria acuminata	Cp4906	19.3206898	0.00031279	
Achromobacter xylosoxidans	Cp4906	16.0471637	0.00159405	
Shinella sp. HZN7	Cp4906	16.6511949	0.00159405	
Devosia soli	Cp4906	15.8199004	0.01715739	
[Desulfobacterium] catecholicum	Cp4906	21.8611511	0.02144033	
Lacrimispora saccharolytica	Cp4906	15.6247841	0.0300987	
Ancylothrix terrestris	Cp4906	20.2111259	0.0321836	

Table S3. Log2 fold changes (Log2FC) of microbial abundance mean from unfertilized soil in peas. Log FC was calculated by subtracting the baseMean counts of log ratios of each microbial taxa at the species level; FDR< 0.05. Enrich group represents the plant variety in which the corresponding bacteria species is in greater abundance. padj represents the FDR adjusted *p*-value.

Peas Vail vs Seed Ranch

Bacteria	Enrich group	Log2 fold changes (Log2FC)	padj	Function
Oscillatoria nigro-viridis	Vail	22.67377895	2.16E-28	*phoD*
Cyanothece sp. PCC 7425	Vail	20.00586773	7.99E-12	*phoD*
Cylindrospermum stagnale	Vail	21.13468721	2.71E-10	*phoD-phoA-phO*
Aliterella antarctica	Vail	17.69294472	9.70E-06	
Oscillatoria acuminata	Vail	18.93349287	8.71E-05	
Devosia soli	Vail	18.44770926	0.000320899	
Shinella sp. HZN7	Vail	15.78658917	0.001388875	
Microcoleus sp. PCC 7113	Vail	7.560223731	0.039649266	
Devosia riboflavina	Vail	15.41359814	0.039649266	

**Bacteria**	**Enrich group**	**Log2 fold changes (Log2FC)**	**padj**	**Function**
Pseudomonas sagittaria	Melrose	-21.088705	1.44E-09	*PSB*
Anaerotaenia torta	Vail	20.5495423	5.42E-09	*phoA*
Achromobacter xylosoxidans	Vail	21.385333	4.74E-08	*PSB*
Rhizobium zeae	Vail	18.0145881	5.84E-06	*phoD*
Lacrimispora saccharolytica	Vail	21.0649017	3.52E-05	
Devosia soli	Vail	18.3548226	0.00033392	
Shinella sp. HZN7	Vail	15.6868557	0.00146209	
Microbacterium foliorum	Vail	20.205793	0.00205167	
Ruminiclostridium hungatei	Vail	22.0841943	0.00440001	
Paenibacillus agaridevorans	Vail	2.81284512	0.02627986	
Devosia riboflavina	Vail	15.3161224	0.03026133	
